# Research progress of migrasomes: from genesis to formation, physiology to pathology

**DOI:** 10.3389/fcell.2024.1420413

**Published:** 2024-08-14

**Authors:** Hua Tang, Zhe Huang, Ming Wang, Xingzhao Luan, Zengfu Deng, Jian Xu, Wei Fan, Dongsheng He, Chong Zhou, Liangbin Wang, Jun Li, Fanfeng Zeng, Dongbo Li, Jie Zhou

**Affiliations:** ^1^ Department of Neurosurgery, The People’s Hospital of Jianyang City, Chengdu, China; ^2^ Department of Neurosurgery, The Affiliated Hospital of Southwest Medical University, Luzhou, Sichuan, China; ^3^ Sichuan Cliniccal Research Center for Neurosurgery, Luzhou, China; ^4^ Laboratory of Brain Function, Southwest Medical University, Luzhou, Sichuan, China; ^5^ Department of Neurosurgery, The Affiliated Hospital of Panzhihua University, Panzhihua, Sichuan, China

**Keywords:** migrasomes, cell migration, TSPANs, cancer, extracellular vesicles

## Abstract

Migrasomes are recently identified organelles that form at the ends or forks of retraction fibers (RFs) behind migrating cells and are expelled from the cell through cell migration. Migrasomes contain signaling molecules which are captured by surrounding cells along with migrasomes or released into the extracellular environment following the rupture of the migrasomes. Finally, through the action of these signaling molecules, migrasomes facilitate the entire process of information conveyance. In addition, migrasomes also serves as a “scavenger” by removing damaged mitochondria from the cell to ensure cellular viability. Thus, migrasomes play a pivotal role in the integration of temporal, spatial, specific chemical information and the clearance of cellular harmful substances, critical for grasping migrasomes’ functions. This review delves into the latest advancements in migrasomes research, covering aspects such as migrasomes’ discovery, distribution, structure and characteristics, genesis and regulation mechanisms, and their correlation with diseases. Additionally, we scrutinize the present investigational findings on migrasomes within the cancer domain, examining their potential impact on cancer and prospective research avenues.

## 1 Introduction

**FIGURE 1:**
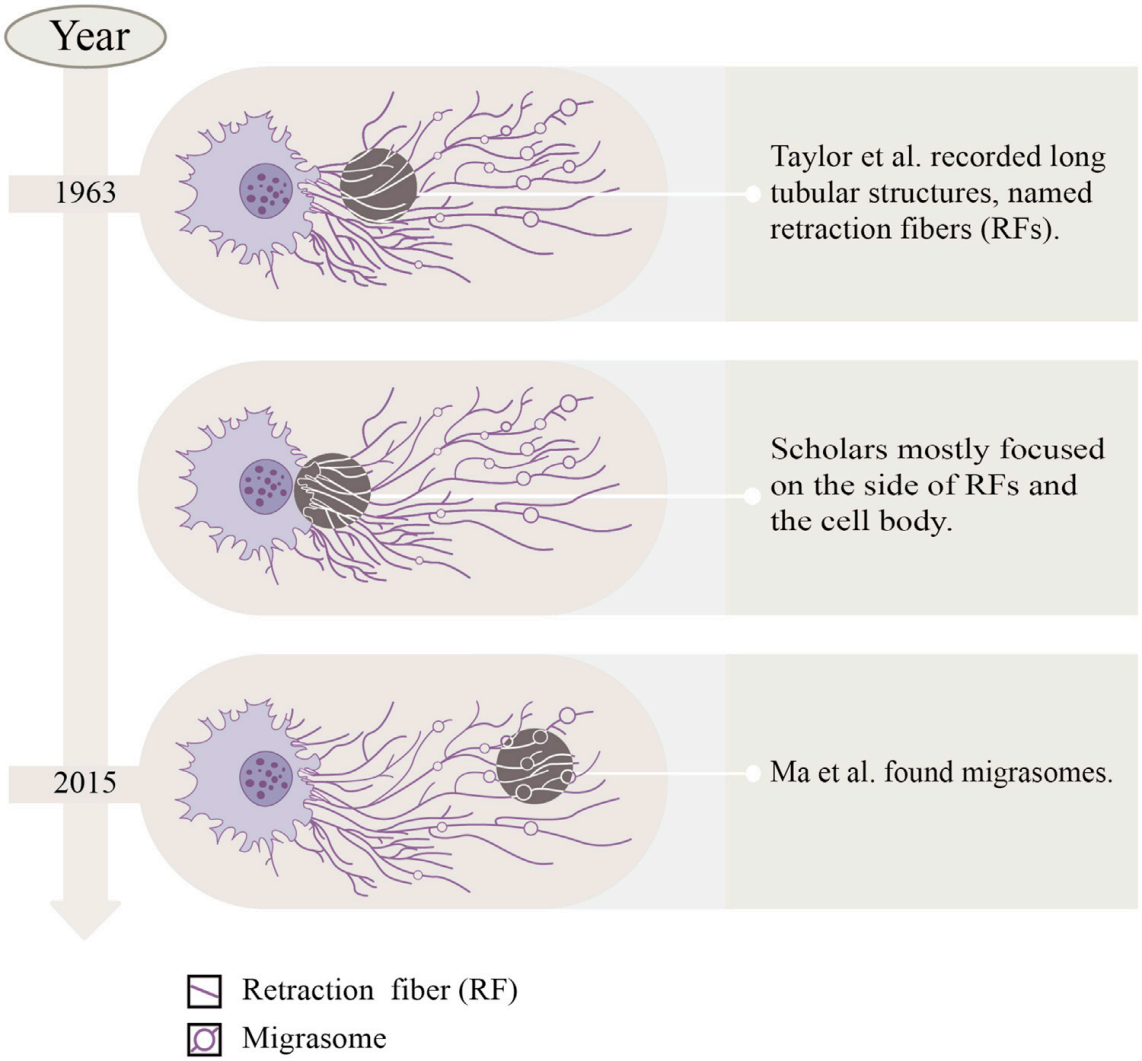
The discovery process of migrasomes.

In the last century, Taylor and Robbins performed optical microscopy (OM) and transmission electron microscopy (TEM) studies, and they recorded the appearance of long tubular structures, named retraction fibers (RFs), when migrating cells retracted from the matrix (Taylor and Robbins, 1963). After that, scholars mostly focused on the side of RFs and the cell body, but ignored the side away from the cell body. Until 2015, Ma et al. (2015) focused on the latter, observing the migration process of normal rat kidney (NRK) cells by TEM, and found a new organelle at the ends or forks of RFs—migrasomes for the first time (Refer to Figure 1). As time went by, migrasomes have been found in many cells or tissues (Refer to Table 1). Recent studies have unveiled that migrasomes are involved in several cellular activities, including intercellular communication (Jiang et al., 2023), immune adjustment (Hyun et al., 2012; Lim et al., 2015; Di daniele et al., 2022; Wang et al., 2022; Li et al., 2023a), lateral transfer of material between cells (Zhu et al., 2021) and mitochondrial quality-control process (Jiao et al., 2021), while also playing a crucial role in influencing tumor dynamics and cancer progression (Deng et al., 2024).

**TABLE 1:** Distribution of migrasomes.

Species	Tissue	Cell
Human	Brain, eye, lung, kidney, bone marrow, et al. (Ma et al., 2015; Schmidt-pogoda et al., 2018; Liu et al., 2020; Jiao et al., 2021; Wu et al., 2022; Li et al., 2023a; Deniz et al., 2023)	MDA-MB231, SKOV-3, HCT116, SW480, MGC803, MIACaPa-2, HaCaT, HPC, NHDF, BM-MSC, Neutrophil, RPE, HUV-EC-C, et al. (Ma et al., 2015; Schmidt-pogoda et al., 2018; Liu et al., 2020; Jiao et al., 2021; Saito et al., 2021; Wu et al., 2022; Li et al., 2023a; Deniz et al., 2023)
Mouse	Brain, kidney, lung, heart, gut, et al. (Ma et al., 2015; Schmidt-pogoda et al., 2018; Liu et al., 2020; Li et al., 2023a; Zheng et al., 2023a; Hu et al., 2023)	L929, B16, HT22, mESC, MEF, RAW 264.7, NIH3T3, et al. (Ma et al., 2015; Liu et al., 2020; Lampiasi et al., 2021; Zhu et al., 2021; Hu et al., 2023)
Rat	Eye, lung, gut, et al. (Ma et al., 2015; Wu et al., 2022; Zhao et al., 2022; Ding et al., 2023)	NRK (Zhao et al., 2022)
Zebrafish	—	Gastrulation (Jiang et al., 2019)
Chick embryo	—	Monocyte (Zhang et al., 2022)

Annotation: MDA-MB231 (breast cancer), SKOV-3 (ovarian adenocarcinoma), HCT116 (colon cancer), SW480 (colon adenocarcinoma), MGC803 (gastric cancer), MIACaPa-2 (pancreatic cancer), HaCaT (keratinocyte), HPC (human podocyte), NHDF (Normal Human Dermal Fibroblasts), HUV-EC-C (Human umbilical vein endothelial cells), BM-MSC (bone Marrow Mesenchymal Stem Cells), Neutrophil, RPE (retinal pigmented epithelium), L929 (mouse fibroblast cell), B16 (Mouse melanoma), HT22 (mouse hippocampal neuronal cell line), mESC (mouse embryonic stem cells), RAW 264.7 (mouse macrophages), MEF (mouse embryonic fibroblast), NIH3T3 (mouse embryo fibroblasts).

## 2 The structure and characteristics of migrasomes

Migrasomes, whose formation depends on cell migration, are a kind of new organelle and extracellular vesicle. Migrasomes are mostly oval in shape, 500–3,000 nm in diameter, and contain many smaller vesicles inside, similar to pomegranate. The number of these vesicles vary widely, from a few to more than 300 (Ma et al., 2015). What are these vesicles? How do they form? What do they do? And are they a special kind of exosomes? There is no definite conclusion at present. As new extracellular vesicles, migrasomes are different from other extracellular vesicles (such as exosomes and microvesicles) in terms of size, origin, lifecycle, formation mechanism, composition and so on (Refer to Table 2). It is important to note that the immobility of migrasomes is determined by the pairing of integrins and the extracellular matrix (ECM) during migrasomes formation. Indeed, Schmidt-Pogoda A et al. showed by proteomic analysis that the migrasome is mainly composed of contractile proteins, actin, myosin, cytoskeleton, and annexin (Schmidt-pogoda et al., 2018). Zhang et al. further found that among the 33 known mammalian tetraspanins (TSPAN, a member of the transmembrane four superfamily), there were 24 TSPANs localizing on migrasomes. However, only 14 TSPANs overexpression could promote the formation of migrasomes. Nine of them (TSPAN1/2/4/6/7/9/18/27/28) showed strong effects (Zhang, 2021). TSPAN4/7, cholesterol, sphingomyelin, integrin α1/α3/α5/β1 are present on migrasomes’ membranes, and they are key molecular markers of migrasomes’ membranes (Ma et al., 2015; Wu et al., 2017; Huang et al., 2019; Liang et al., 2023). Significantly, these markers are also present in other extracellular vesicles such as exosomes (Zhang et al., 2019). Zhao et al. identified migrasome-specific markers by quantitative mass spectrometry and found that NDST1, PIGK, CPQ, and EOGT proteins were enriched in migrasomes but rarely detected in other extracellular vesicles. To make it easier to understand, we have summarized the roles of the proteins and markers associated with migrasomes in Table 3. In addition, they tested these four markers in serum samples and the results showed the presence of migrasomes in serum, which could open avenues for translational research on migrasomes in the diagnosis, prognosis, and therapeutic applications of related diseases (Zhao et al., 2019a). There are still questions to be answered. For example, which cells produce serum-migrasomes? Where are these serum-migrasomes produced? How do these migrasomes enter the blood circulation system? And what are their functions? We speculate that these serum-migrasomes may be generated by some cells during the process of crossing the vascular endothelium (e.g., tumor metastasis through blood vessels, and monocyte chemotaxis through blood vessels, etc.). So their functions may be related to their origin. Of course, more researches are needed to verify our conjectures.

**TABLE 2:** Comparison of migrasomes and exosomes.

	Migrasomes	Exosomes	Microvesicles
Essence	Organelle	Extracellular vesicle	Extracellular vesicle
Origin	Migrating cell	Multivesicular bodies (MVB)	Plasma membrane
Diameter	500–3,000 nm	50–150 nm	100–1,000 nm
Shape	Mostly oval	Spherical or oval	Irregularly spherical or oval
Lifecycle	About 400 min	A few minutes to a few hours	A few minutes to a few hours
Formation mechanism	Formed by the assembly of large domains on the migrating cytoplasmic membrane	Multivesicular bodies (MVBs) generate vesicles and fuse with the plasma membrane to release exosomes	Formed by regulated release of outward budding of the plasma membrane
Marker	TSPAN 4/7, cholesterol, integrin α5/β1, WGA, NDST1, PIGK, CPQ, EOGT	CD9, CD63, CD81, HSP70, HSP90, ALIX, TSG101	CD40, flotillin-2, selectins, integrins, metalloproteinases, phosphatidylserine
Content	Proteins, RNAs, damaged organelles, numerous smaller vesicles	Proteins, RNAs, DNAs, cholesterol	Proteins, RNAs, DNAs,cholesterol
ECM-dependent	Yes	No	No
References	Ma et al. (2015), Wu et al. (2017), Chen et al. (2019), Huang et al. (2019), Jiao et al. (2021), Zhu et al. (2021), Lee et al. (2024)	Lindenbergh and Stoorvogel (2018), Dai et al. (2020), Guillamat-Prats (2021)	Jayachandran et al. (2012), Sedgwick and D’souza-schorey (2018), D’souza-schorey and Clancy (2012)

**TABLE 3:** The proteins and markers associated with migrasomes.

Name	Role in migrasomes
TSPAN4	Interacts with cholesterol to form microdomains (TEMs) that facilitate the growth of migrasomes (Huang et al., 2019)
Integrin α5/β1	Enriched at the bottom of migrasomes and anchors migrasomes to the ECM (Wu et al., 2017)
NDST1	Unknown
PIGK	Unknown
CPQ	Unknown
EOGT	Unknown

## 3 Mechanisms of migrasomes genesis and regulation

**FIGURE 2:**
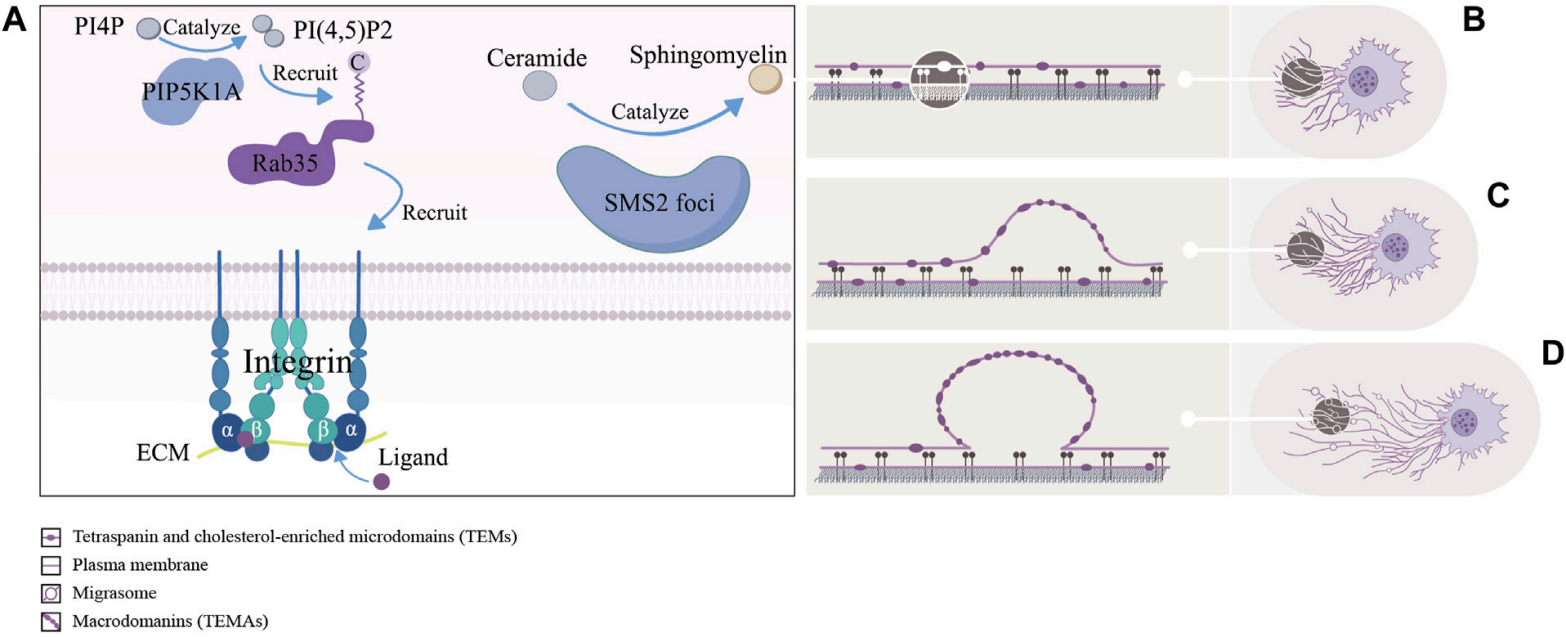
The left picture **(A)** shows a schematic representation of the formation site of migrasomes: including the PIP5K1A-Rab35 axis pairs with the ECM by recruiting integrin α5/β1, and sphingomyelin synthase 2 (SMS2) synthesizes ceramides to sphingomyelins (SM) at the SMS2 foci. The right picture **(B–D)** shows approximate process of formation of migrasomes: as the cells migrate, tetraspanin and cholesterol-enriched microdomains (dark purple ovals) gradually assemble into macrodomains on retracting fibers, which increase the bending stiffness of the membrane beyond that of other regions, thereby driving migrasomes formation.

During migration, cells leave RFs at the tail. At the ends and forks of RFs, migrasomes with a diameter of 500–3000 nm were formed by localized swelling of the plasma membrane. Thereafter, as the cells continued to migrate, the RFs connecting the cell bodies gradually become thinner and finally break, and the migrasomes on them were exposed into the surrounding matrix, captured by the surrounding cells or ruptured to release their contents (Ma et al., 2015). Undoubtedly there were a variety of factors to regulating the formation of migrasomes (Refer to Figure 2), and we collected and collated recent studies, as shown below:

Through immunofluorescence assays, Ding et al. found that phosphatidylinositol 4-phosphate 5-kinase (PIP5K1A), a kinase that converts PI4P to PI(4,5)P2, was recruited to the site of migrasomes formation, and PI(4,5)P2 was generated at this site. Once PI(4,5)P2 accumulated, it recruited Rab35 to the site of migrasomes formation by interacting with the C-terminal multibase cluster of Rab35. And activated Rab35 promoted migrasomess formation by recruiting integrin α5 at the migrasomess formation site. Notably, time-lapse imaging experiments suggested that the enrichment of PIP5K1A, PI(4,5)P2, Rab35 and integrin α5 in migrasomes was earlier than that of TSPAN4 (Ding et al., 2023). This implied that these proteins were functional in the early migrasomes. However, Wu et al. reported that integrin α5 and β1, mainly enriching at the bottom of migrasomes, adhered migrasomes to ECM by pairing with the ECM and promoted the formation of migrasomes (Wu et al., 2017). These studies suggested that the PIP5K1A-Rab35 axis was fixed at the migrasomes formation site through the pairing of integrin α5 with ECM. But it was unclear how exactly PIP5K1A was recruited to the site of migrasomes formation.

Liang et al. found that sphingomyelins (SM), synthesized from ceramides by sphingomyelin synthase 2 (SMS2) in the plasma membrane, were necessary for the growth and maintenance of migrasomes. Notably, the enhancement of NT-Lys signal, a sphingomyelin marker, was faster in the early stages of migrasomes formation compared to TSPAN4-GFP. For further study, they carried out the live cell imaging analysis experiments and found two sphingomyelin synthase 2 (SMS2^+^) structural pools in migrating cells. One consisted of larger TSPAN4^+^ structures in the perinuclear region and the other consisted of smaller TSPAN4^−^ puncta. The former was located in the cytoplasm, while the latter was distributed in the leading edge of the basal membrane and formed the SMS2 foci. Once formed the SMS2 foci become immobile on the surface that contacted the cell. When the cell moved away, these SMS2 foci were left behind on RFs and grow into migrasomes. Confusingly, immunofluorescence experiments showed that the SMS2 foci were not co-localized with integrins α5 and β1 (Liang et al., 2023). In addition, previous studies had shown that sphingomyelin and cholesterol tend to spontaneously form ordered lipid molecular domains with tight aggregation and reduced mobility, which might be related to the recruitment of cholesterol at the migration formation site (Sezgin et al., 2017). Liang et al.’s works showed that SM and SMS2 were both key factors in the early formation of migrasomes, and the concept of SMS2 foci enriched the content of the formation site of migrasomes. It was unclear if SMS2 foci only form at the leading edge of the basal membrane, however because of the lack of colocalization with integrins α5 and β1, they must had a different attachment mechanism. Meanwhile the relationship between the PIP5K1A-Rab35 axis, participated in the assembly of the formation site of migrasomes through integrin α5, and SMS2 foci at this site was worth studying.

Ma et al. treated cells with cytoslatin B and Latrunculin A (two actin polymerization inhibitors) and CK636 (an inhibitor of the Arp2/3 complex that blocks the formation of branching actin networks) and found that these inhibitors reduced the number of migrasomes by preventing the formation of migrasomes. It suggested that actin polymerization might be necessary for the formation of migrasomes, either by influencing cell migration or directly involved in migration biogenesis (Ma et al., 2015). Through the live cell imaging experiment Fan C et al. found that when L929 did not continuously migrate to a certain direction during rotation, the formation of RFs and migrasomes was reduced. In addition, when the cell migration was longer and faster, more migrasomes were formed (Fan et al., 2022). These studies suggested that the directional and continuous migration of cells were a necessary condition for the formation of migrasomes, and the speed of migration was also one of the factors affecting the formation of migrasomes.

Using live-cell imaging techniques and biomimetic systems of migrasomes and RFs, Raviv Dharan et al. found that migrasomes were formed in two stages. In the first stage, also known as the rapid initial stage, researchers hypothesized the local swelling of the RFs that formed early migrasomes was driven by membrane mechanical stress. In the second phase, also known as the stable phase, TSPAN4 was recruited to stabilize and promote the maturation of the early migrasomes. In addition, researchers also found that the number of early and mature migrasomes both increased significantly when TSPAN4 was overexpressed, but only the number of mature migrasomes decreased observably after TSPAN4 knockout (Dharan et al., 2023). These results suggest that the formation of early migrasomes is primarily influenced by membrane mechanical stress, whereas mature migrasomes are predominantly influenced by TSPAN4. It is well-established that tetraspanin proteins form tetraspanin and cholesterol-enriched microdomains (TEMs) on membranes (Le naour et al., 2006; Zuidscherwoude et al., 2015). Huang et al. found that at the end of the stage of migrasomes formation, TSPAN4 was gradually recruited to the sites of migrasomes formation and assembled into TSPAN4 spots. Furthermore, fluorescence recovery after photobleaching (FRAP) assays demonstrated that once TSPAN4 is recruited to the migrasomes, it cannot move out. To explore the dynamics of TEMs assembly into macrodomains (TEMAs), they they developed an *in vitro* migrasome formation system. By manually pulling the GUV membrane through applying direct force to the TSPAN4 spots, they induced the formation of stretched membrane tethers simulating the RFs. The pulling caused the tethers to lengthen and narrow, facilitating the self-organization of initially uniformly distributed TSPAN4-rich TEMs into TEMAs, which subsequently swelled to form migrasomes. Additionally, confocal microscopy observations of NRK cells revealed that TEMs are cholesterol-rich, and that cholesterol depletion impedes migrasome formation. Finally, through theoretical deduction, Huang et al. demonstrated that these TEMAs increase the bending stiffness of the membrane beyond that of other regions, thereby driving migrasome formation (Huang et al., 2019). This finding supports the hypothesis proposed by Raviv Dharan et al. that membrane mechanical stress is a key driver of migrasome formation. However, the mechanisms behind TSPAN4’s recruitment to the migrasome formation sites, and its inability to return to the RFs once recruited, remain unexplained.

## 4 Function of migrasomes

**FIGURE 3:**
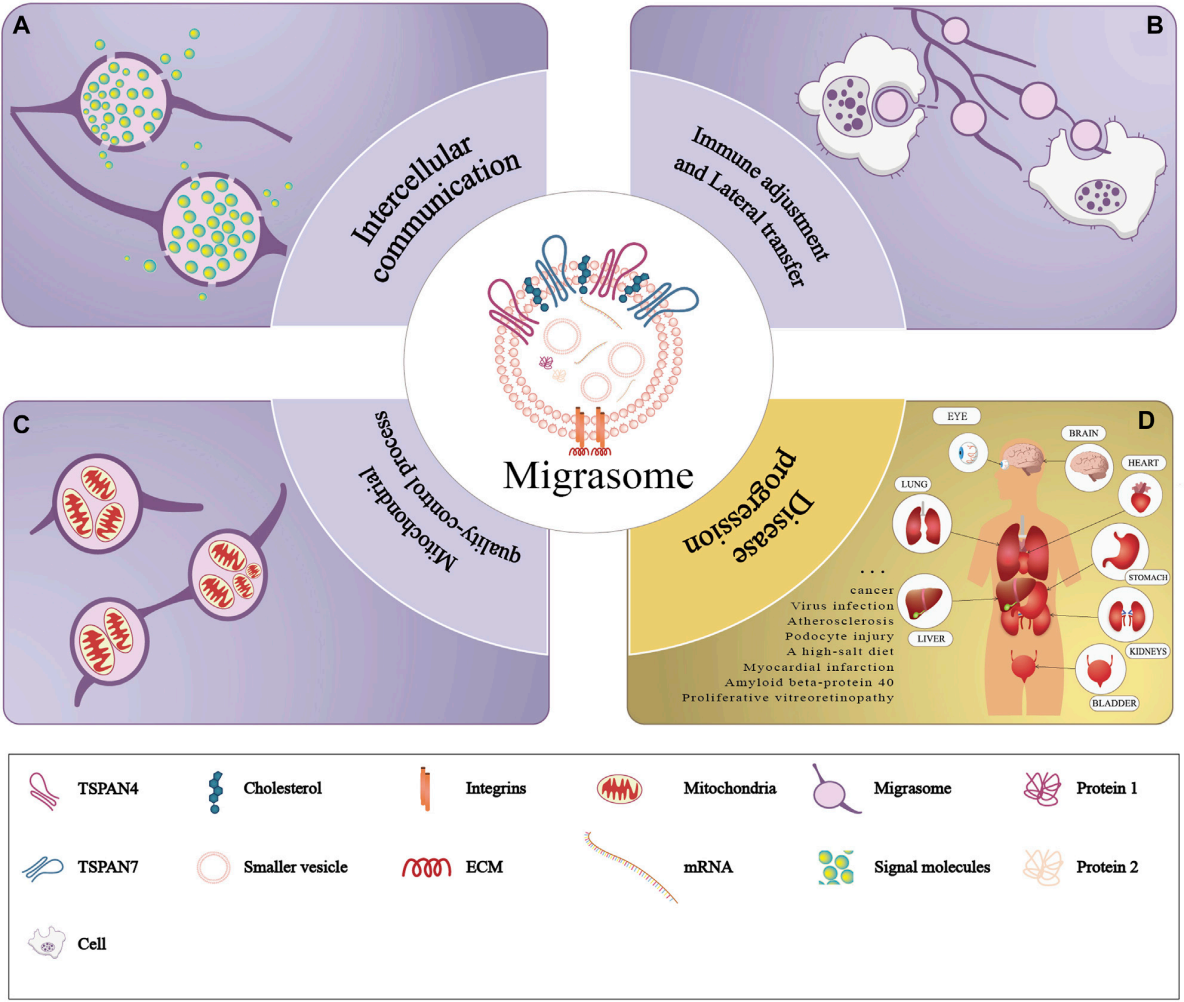
Functions of migrasomes. The purple part of the figure represents three modes of physiological functions of migrasomes: **(A)** migrasomes release their contents in the extracellular matrix (intercellular communication), **(B)** the migrasomes and their contents are captured by the other cells (immune adjustment and lateral transfer), and **(C)** migrasomes dispose of damaged mitochondria (mitochondrial quality-control process). The yellow part of the figure **(D)** represents pathology of migrasomes including migrasome-associated diseases and tissue distribution.

Recent studies have unveiled that migrasomes are involved in various fields including intercellular communication, immunoregulation, lateral transfer of material between cells and mitochondrial quality-control process. We review previous studies to summarize the currently known function of migrasomes (Refer to Figure 3).

### 4.1 Migrasomes and intercellular communication

#### 4.1.1 Migrasomes regulate embryonic development

Jiang et al. found a large number of migrasomes in zebrafish embryos. Carring out tandem-mass-tag (TMT) labeling followed by quantitative mass spectrometry analysis, they found that these migrasomes were enriched in chemokines, morphogens and growth factors, such as CXCL12. Whlie in the CXCL12a^−/−^ model fish, researchers observed the impairment of organ morphogenetic, which was effectively rescued by injected the purified migrasomes from WT gastrulas into into CXCL12a morphant embryos. Researchers also found that CXCL12 in migrasomes interacted with its ligand CXCR4 expressed on dorsal forerunner cells (DFCs) to induce chemotaxis and recruitment of DFCs, thereby ensuring proper localization of DFCs and subsequent organ morphogenesis (Jiang et al., 2019).

#### 4.1.2 Migrasomes promote angiogenesis

Zhang et al. repoted highly migratory monocytes appeared at the area of capillary formation in chick embryo chorioallantoic membrane and found numerous migrasomes along their migratory trajectories. Quantitative mass spectrometry showed that monocyte migrasomes were enriched with pro-angiogenic factors and chemokines, such as VEGFA and CXCL12. And monocytes deposit migrasomes enriched in pro-angiogenic factors and chemokines to promote angiogenesis and monocyte recruitment *in vivo* (Zhang et al., 2022).

#### 4.1.3 Migrasomes promote tumor cells recruitment

Deniz IA et al. found that bone marrow mesenchymal stromal cell-associated migrasomes attracted leukemia cells (KG-1a) by releasing chemoattractants such as SDF-1, and the mechanism was involved in the selective localization of KG-1a cells by mediating the CXCR4-SDF-1 axis (Deniz et al., 2023). This represented that under pathological conditions such as cancer, tumor cells might usurp the normal material or information exchange between healthy cells, leading to a shift in the cellular microenvironment that promotes cancer growth and metastasis.

### 4.2 Migrasomes and immune adjustment

With liquid chromatography-tandem mass spectrometry (LC-MS/MS), Li et al. found that bone marrow mesenchymal stem cells (BM-MSC) load the antibacterial peptide dermcidin (DCD) in migrasomes upon bacterial stimulation. *In vivo* tracking experiment showed that when BM-MSC were transplanted into a mouse model of 60 min of transient middle cerebral artery occlusion (tMCAO), these cells quickly penetrated into the lungs and disappeared within 24 h, leaving migrasomes enriched in the antimicrobial peptide dermcidin (DCD) to enhance macrophage LC3-associated phagocytosis (LAP) and improve bacterial clearance (Li et al., 2023a). Hyun et al. identified membrane-covered structures approximately 1 μm in diameter in the migration path of neutrophils. This membrane-covered structures also released CXCL12 and directed T cells to move along the path of neutrophil migration (Hyun et al., 2012). In contrast, Lim et al. showed that depletion of neutrophil-derived CXCL12 and antagonism of CXCR4 abolished neutrophil guidance of T cell migration (Lim et al., 2015). Di Daniele et al. showed that migrasomes released from tumor cells may facilitate cancer cells to evade the immune system by inducing apoptosis of effector T cells, inhibiting proliferation of natural killer cells, or inducing differentiation of monocytes into immunosuppressive macrophages (Di daniele et al., 2022). Moreover, Wang et al. found that PD-L1 was mainly concentrated in the tail of migrating cancer cells, forming PD-L1-rich RFs and migrasomes. When PD-L1-rich migrasomes left the RFs and entered the ECM, these migrasomes might be captured by neighboring cells and increased expression of PD-L1, thereby inhibiting the individual immune response and changing the tumor microenvironment (Wang et al., 2022).

### 4.3 Lateral transfer of material between cells

Zhu et al. found that the migrasomes contained RNA (mainly mRNA) as well as proteins, and RNA-seq showed that these nucleic acids were highly enriched in cellular biological processes related to metabolism, intracellular transport, cell junction, vesicle fusion, as well as assembly of subcellular and membrane structures. For example, they found the presence of the full-length Pten gene in the migrasomes. And when these migrasomes were co-cultured with U87, MDA-MD-468, and PC3 cells, which did not express Pten protein, they found that the cells re-expressed Pten protein. Migrasomes treated with proteinase K, in which Pten protein was completely removed, were co-cultured with recipient cells, then the researchers still detected Pten protein in recipient cells, which meant that Pten mRNA was transferred into recipient cells and translated into protein. This suggested that migrasomes were involved in the lateral transfer of material between cells and had functional effects on recipient cells (Zhu et al., 2021).

### 4.4 Mitochondrial quality-control process

Jiao et al. found that when macrophages or neutrophils were subjected to mild mitochondrial stress, these cells could avoid binding of damaged mitochondria to inward motor protein (dynein) and enhance binding to KIF5B (Kinesin Family Member 5B) through a mitochondrial quality-control process. Finally, the damaged mitochondria were transported into the migrasomes and excreted together, thereby helping to maintain the cell viability of macrophages and neutrophils (Jiao et al., 2021).

## 5 Migrasomes in diseases

### 5.1 Nonneoplastic diseases

#### 5.1.1 Virus infection

One of the classical functions of extracellular vesicles (Evs) is to influence the immune response by transmitting pathogens. So does the migrasomes, a new member of the Evs family, have this function? Lv et al. stained A4-YFP vaccinia virus (VACV)-infected HeLa cells with the migrasomes probe FM4-64 and detected migrasomes-like structures, which suggested that VACV infection may trigger migrasomes formation. To further confirm the induction of migrasomes by VACV, they transfected HeLa or Huh7.5.1 cells with plasmids expressing HA-tagged MPXVgp045 [an abundant viral protein found in intracellular mature viruses, intracellular enveloped viruses, and extracellular enveloped viruses (Vliegen et al., 2012)] and mCherry-tagged TSPAN4 for 24 h and subsequently infected the cells with A4-YFP VACV WR strain for 34 h. Finally, migrasomes-like structures containing MPXVgp045-HA and A4-YFP signals were found under fluorescence microscope, indicating that VACV virions may be located in migrasomes and spread through migrasomes (Lv and Zhang, 2023). In addition, Liu et al. found that herpes simplex virus (HSV-2) may also be transmitted by the migrasomes (Liu et al., 2023). Moreover, it has been shown that after SARS-CoV-2 infection, platelets will internalize the virus, triggering the programmed death of platelets and the release of migrasomes. The contents of these migrasomes may be highly thrombotic or pro-inflammatory, and may lead to disorders of immune activation (Koupenova et al., 2021).

#### 5.1.2 Cardiovascular diseases

Jiao et al. found that migrasomes and TSPANs were closely related to cardiovascular homeostasis, which implied that migrasomes may be involved in cardiovascular diseases (Jiao et al., 2021). Zheng et al. recently found that TSPAN4 expression was highly associated with atherosclerosis regression-related macrophages, intraplaque hemorrhage and ruptured plaques. Moreover, TSPAN4 is upregulated in mouse models of spontaneous myocardial infarction (MI) and induced MI, suggesting that TSPAN4 and migrasomes may be potential targets of macrophages involved in atherosclerosis (Lin et al., 2019; Zheng et al., 2023a; Zheng et al., 2023b).

#### 5.1.3 Urinary diseases

Liu et al. found that during podocyte injury induced by puromycin aminonucleoside (PAN), the secretion of human or murine podocyte migrasomes was strongly increased, which preceded the increase of proteinuria. In addition, an increased number of migrasomes was also detected in the urine of diabetic nephropathy patients with proteinuria level <5.5 g/day (Liu et al., 2020; Ardalan et al., 2022). And Yang, R et al. examined the high levels of migrasomes in the urine of kidney disease (KD) patients with podocyte injury (Yang et al., 2024). These data suggest that migrasomes are more sensitive to podocyte injury and can be used as potential markers of early podocyte injury.

#### 5.1.4 Nervous system diseases

Antje et al. found that a high-salt diet promoted ischemic injury in the CNS and induced the formation of numerous migrasomes in ischemic brain parenchyma. These migrasomes were rich in neuronal debris and often presented near atrophic neurons, indicating that migrasomes may mediate the clearance of damaged neurons or exacerbate neuronal atrophy (Schmidt-pogoda et al., 2018). Moreover, Hu et al. found that amyloid beta-protein 40 (Aβ40) stimulated macrophage lineage cells to overproduce migrasomes containing CD5L, a molecule associated with complement activation, thereby further contributing to complement-dependent blood-brain barrier injury (Hu et al., 2023).

#### 5.1.5 Retinopathy

Wu et al. found that TSPAN4, a migrasome marker, was abundantly expressed in clinical samples related human proliferative vitreoretinopathy (PVR). Moreover, these migrasomes can be internalized by the retinal pigment epithelium (RPE), increasing abilities of migration and proliferation of RPE, thereby promoting the progression of PVR (Wu et al., 2022).

### 5.2 Neoplastic diseases

The invasion and metastasis of cancer cells are the primary causes of mortality in cancer patients. Migrasomes, which are structures formed during the cell migration process, have increasingly become a focus of research due to their relationship with cancer cells. Currently, the study of migrasomes in tumors is still in its early stages, with most research focusing on migrasome markers. For example, Zheng et al. found that TSPAN4 was highly correlated with tumor-associated macrophages (Zheng et al., 2023a). Furthermore, Qing et al. utilized pan-cancer analysis and single-cell sequencing to find that genes associated with migrasomes are highly correlated with immune evasion and the tumor microenvironment, indicating that these genes were potential immunotherapy targets, and their high expression predicted to poor prognosis (Qin et al., 2022). Qi et al. found that the expression levels of TSPAN4 in gastric cancer tissues were significantly higher than in adjacent non-tumor tissues. They demonstrated that both *in vivo* and *in vitro*, downregulation of TSPAN4 could inhibit the growth of gastric cancer cells, suggesting that TSPAN4 might play a role in slowing the progression of gastric cancer (Qi et al., 2018). Zhao et al., through genome-scale CRISPR activation screening, identified TSPAN4’s association with chemoresistance in esophageal tumors. Overexpression of TSPAN4 in esophageal squamous cell carcinoma (ESCC) cell lines notably promoted resistance to paclitaxel (PTX) by inhibiting cell apoptosis (Zhao et al., 2019b). Using real-time quantitative reverse transcription PCR (RT-PCR), Western blot analysis, and immunohistochemical analysis of liver cancer (HCC) tissue microarrays, Li et al. reported that mRNA and protein levels of TSPAN4 were elevated in about 80% of HCC tissues. Additionally, knocking down TSPAN4 significantly inhibited the proliferation of liver cancer cells (Li et al., 2012).

Using enrichment pathway analysis, protein-protein interaction analysis, immunophenotype and pan-cancer analysis, Zheng et al. found that TSPAN4 expression was associated with cancer, especially in glioblastoma multiforme and low-grade gliomas (Zheng et al., 2022). Yu et al. found that TSPANs were highly correlated with the prognosis and drug resistance of glioma through bioinformatics analysis. And western blot experiment, scratch experiment, transwell experiment and plate cloning experiment showed that the expression level of TSPAN4 protein in U251, U87 and T98G cells was higher than that in normal cells. In addition, knockdown of TSPAN4 could inhibit the proliferation, migration and invasion of glioma cells, and immune correlation analysis suggested that TSPANs were related to the formation of tumor microenvironment (TME), which may affect the outcome of immunotherapy (Li et al., 2023b). These evidences suggest that migrasomes may participate in the tumor microenvironment and have the potential to promote the occurrence and development of glioma. Recently, Lee SY et al. showed that fibronectin (FN) -integrin α5β1 interaction can induce the generation of RFs and promote the motility of GBM cells. Notably, they observed RFs and migrasomes behind GBM cells under electron microscopy, which was the first direct evidence of glioma migrasomes (Lee et al., 2021). In a subsequent study, Lee SY et al. found that violently migrating GBM cells formed a retraction fiber and migrasome (R&M) in the tail, which contained autophagosomes inside and could be discharged from GBM cells with the migrasomes. And inhibition of autophagosome/lysosome fusion by chloroquine (CQ) increased the production of migrasomes and alleviated ER stress in GBM cells (Lee et al., 2024). GBM cells avoid death by excrete autophagosomes through migrasomes, which in turn may be captured by surrounding cells to induce autophagy. This membrane material exchange creates a favorable environment for cancer cells.

## 6 Perspective

The formation of migrasomes is a complex process regulated by precise mechanisms. Although in this article we have sorted out the existing studies and teased out the regulatory mechanisms of migrasomess formation, there are still some questions to be explored. For example, what proteins make up the formation site of migrasomes? What factors regulate it? How exactly are TSPAN4 and cholesterol recruited to the migrasomes formation site? As a new type of extracellular vesicle, migrasomes contain a variable number of small vesicles in their interior. What are these small vesicles? When and where did they form? Are they also a new type of extracellular vesicle? Is the material contained within them the material basis for the function of the migrasomes? Are migrasomes the ultimate functional executors or do they act by releasing their internal vesicles?

In addition, migrasomes of different origins have different functions. For example, BM-MSC migrasomes mediate the secretion of antimicrobial peptide dermcidin and enhance macrophage LC3-associated bacterial phagocytosis (LAP) (Li et al., 2023a). And monocyte migrasomes promote capillary formation through VEGFA and CXCL12 (Zhang et al., 2022). This suggests that the different contents of migrasomes of different origins may be the reason for their different functions. This not only provides a broad prospect for the study of migrasomes, but also brings many problems to researchers. Because the research progress of migrasomes from one source may not be representative of all migrasomes. After all, summarizing general rules from special cases is a big challenge. However, migrasomes have great application prospects in organ development, homeostasis maintenance, immune regulation, disease diagnosis and treatment. In the future, more researches are needed to enrich the biological functions of migrasomess.

The formation of migrasomes is highly dependent on cell migration, and tumor cells are often highly migratory, so tumor-associated migrasomes may be generated during the process of tumor cell migration. At present, various bioinformatics studies have suggested that migrasomes are related to pan-cancer (Zheng et al., 2022). Researchers have shown that the migrasome marker TSPAN4 can affect the growth of esophageal cancer (Zhao et al., 2019b), gastric cancer (Qi et al., 2018), glioma (Lee et al., 2021) and liver cancer (Li et al., 2012). Moreover, Lee SY et al. found migrasome-like structures in glioma tissues for the first time under electron microscopy (Lee et al., 2021), and GBM cells can reduce endoplasmic reticulum stress by removing autophagosomes by migrasomes (Lee et al., 2024). Combined with studies that migrasomes are involved in PDL1 transport (Wang et al., 2022), migrasomes promote angiogenesis (Zhang et al., 2022) and migrasomes induce apoptosis of effector T cells (Di daniele et al., 2022), it is suggested that the future research of migrasomes in cancer can focus on these directions. In addition, centrioles can relieve mitochondrial stress by exportating damaged mitochondria through migrasomes (Jiao et al., 2021). In the process of rapid proliferation of malignant tumor cells, the ROS level increases, which inevitably causes mitochondrial stress damage. Can tumor cells reduce mitochondrial stress to maintain cell viability through the removal of damaged mitochondria by migrasomes? Migrasomes have been found to be capable of lateral transport of substances (Zhu et al., 2021), so what is the role of lateral transport between tumor and normal cells in glioma? What is the role of lateral transport between tumor cells? Is there a correlation between tumor heterogeneity and lateral transport? These questions deserve further study.
